# A Perspective of Magnetoelectric Effect in Electrocatalysis

**DOI:** 10.1002/smsc.202300065

**Published:** 2023-08-13

**Authors:** Dongsheng Shao, Tianze Wu, Xiaoning Li, Xiaoming Ren, Zhichuan J. Xu

**Affiliations:** ^1^ School of Materials Science and Engineering Nanyang Technological University Singapore 639798 Singapore; ^2^ State Key Laboratory of Materials-Oriented Chemical Engineering and College of Chemistry and Molecular Engineering Nanjing Tech University Nanjing 211816 China; ^3^ The Centre of Advanced Catalysis Science and Technology Nanyang Technological University Singapore 639798 Singapore

**Keywords:** electrocatalysis, magnetic fields, magnetoelectric effects, multiferroic materials

## Abstract

The integration of magnetic fields with magnetoelectric (ME) coupling materials has been recently reported for electrocatalysis applications. Highly efficient energy conversion and storage can be potentially provided by this emerging approach. The ME properties, that is, the coexistence of ferromagnetic (FM) and ferroelectric (FE) ordering in some multiferroic materials, can be manipulated by magnetic or electric fields. The ME coupling can result in unique spin‐related physical properties in catalysts, further leading to interesting effects on electrocatalytic reactions. Herein, a discussion on the ME coupling multiferroic materials, as well as their potential opportunities and challenges as electrocatalysts in selected electrochemical reactions, is provided.

## Graphical abstract

The electronic structure of magnetoelectric coupling materials can be tuned by applying an external magnetic or electric field, which can result in enhanced electrocatalytic performance during electrochemical reaction.

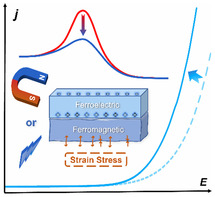

## 1 Introduction

As industrialization has progressed over the centuries, energy demand has increased greatly. Unfortunately, the overconsumption of nonrenewable fossil fuels leads to an unsustainable energy economy and causes detrimental effects on the environment. It is crucial to develop energy technologies to convert and store the renewable energy generated by solar, wind, hydropower, etc. Electrocatalysis plays an essential role in those energy conversions and storage technologies^[^ ^1^ ^]^ and its advances rely on the development of efficient catalysts and electrode materials. Recent studies have revealed that the chemical and physical characteristics of some functional catalysts can be tuned by external factors for promoting electrocatalytic performance. For example, the external stimuli, including thermal, stress, electricity, and magnetism^[^ ^2^ ^]^ can trigger new physical and chemical characteristics of catalyst materials to assist catalysis reactions, such as dielectric responses induced by thermotropic phase transitions,^[2c]^ piezochromism,^[2d]^ electrostriction,^[^ ^3^ ^]^ and FM or FE moment order.^[^ ^4^ ^]^ The activated additional functions of catalysts not only have great promise to prompt their catalytic performance but also provide more opportunities for fundamental mechanism investigation. Inspired by these findings, more efforts are being devoted to improving the catalytic performance by applying external magnetic fields to the catalysts.

The effects of an external magnetic field on electrochemical reactions have been studied since the 1990s.^[^ ^5^ ^]^ The working mechanisms were found complicated, but most of them are associated with the influence of mass transportation near the electrode surface. We have summarized these phenomena in a recent review article.^[^ ^6^ ^]^ Different from the findings reported in the 1990s, some recent studies including ours have found more additional effects of an external magnetic field on the catalytic activity for the ferromagnetic catalysts. For instance, we have investigated several model catalysts and revealed the role of spin polarization in the oxygen evolution reaction (OER) under a magnetic field,^[^ ^7^ ^]^ which is believed to reduce the energetic barrier toward the formation of ground‐state oxygen, triplet oxygen (^↑^O=O^↑^).^[^ ^8^ ^]^ The OER is a four‐step reaction and each step is accompanied with one electron transfer. Our findings indicate that the electron transfer step to form an M–O· intermediate is a spin‐polarization process, which is sensitive to spin regulation by a magnetic field. Besides the influence of magnetic polarization, it should be noted that a magnetic field can also induce intriguing electric polarization in the magnetoelectric (ME) coupling materials.^[^ ^9^ ^]^ ME effect is characterized by the appearance of an electric polarization tempered by a magnetic field or vice versa, which has been extensively investigated in the fields of information storage, advanced logic devices, sensors, and so on. The magnetic field induced electric polarization inside ME electrocatalysts could bring about positive effects on the electrical catalysis, as in this case electrons will not only be driven by electricity from the power supply but also influenced by electric polarization. On the contrary, the electric field generated inside an ME material introduced by the electric power supply could also alter its magnetic polarization, which will regulate electron behaviors during the reactions. Thus, multiple responsive ME materials should have larger freedom of regulation under the magnetic field, given their more distinctive physical properties than single‐responsive ferromagnetic (FM) or ferroelectric (FE) materials.^[9b]^ To date, some emerging studies have indicated that the ME coupling materials can affect the catalytic performance of an electrochemical reaction such as OER and the hydrogen evolution reaction (HER).^[^ ^10^ ^]^

In this perspective, we discuss the influence of magnetic fields on electrocatalytic processes when ME coupling materials are involved. The fundamentals of the ME effect in multiferroic materials are briefly introduced. Recent breakthroughs in using the ME effect in electrocatalysis are discussed. Based on the current research, we also provide perspectives on the potentials and challenges of applying ME effects in electrocatalysis.

## 2 Magnetoelectric Coupling in Multiferroic Materials

The common feature of ME coupling materials is that they possess simultaneous magnetic and electric orderings in a material, as well as the capability to exhibit changes in electric polarization (*P*) in response to a magnetic field (*H*), in magnetization (*M*) in response to an applied electric field (*E*). According to the definition, the ME coupling is usually realized in some multiferroic materials. Multiferroics are a class of materials that simultaneously exhibit multiple ferro‐orderings such as FM, FE, and ferroelastic properties.^[^ ^11^ ^]^ The ME coupling is observable when the FM and FE orderings are coupled, whether it is a single phase or a composite material. The ME coupling arises from the interaction between the magnetic and electric orderings due to the spin–orbit coupling in a single phase.^[^ ^12^ ^]^ It also depends on the lattice strain and interfacial electronic reconstruction (orbital–charge coupling) in a composite.^[^ ^13^ ^]^ The strength of ME coupling is evaluated using the second‐order ME susceptibility tensor, represented by the ME coupling coefficient *α*_ME_ in the following equations.^[9a]^

(1) $$P=\alpha_{\text{ME}}H$$

(2) $$M=\;\alpha_{\text{ME}}E$$

(3) $$\text{ME effect}=\frac{\text{Magnetic}}{\text{Mechanical}}\times \frac{\text{Mechanical}}{\text{Electric}}$$

Unfortunately, single‐phase multiferroic materials with simultaneous FE and FM ordering are even rare at room temperature, while fabricating these with high ME coupling coefficient *α*_ME_ is more challenging. The earliest reported single‐phase multiferroic materials such as Cr_2_O_3_, and Na_0.5_Bi_0.5_TiO_3_^[^ ^14^ ^]^ have small *α*_ME_ due to the fact that the difference in filling of the d‐orbitals required for magnetism and ferroelectricity makes these two ordered states mutually exclusive.^[^ ^15^ ^]^ Currently, the *α*_ME_ of most single‐phase multiferroic materials is not high at room temperature, except for BiFeO_3_, Bi_5_Ti_3_FeO_15_, and SrCo_2_Fe_24_O_41_,^[^ ^16^ ^]^ giving difficulties to meet the demands in practical applications.^[^ ^17^ ^]^

**Figure 1:**
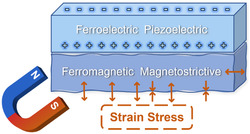
The schematic representation of ME effect in magnetostrictive/piezoelectric bilayer under a magnetic field.

To address this, composite ME materials with two phases (FM/FE) proposed in 1970^[^ ^18^ ^]^ have been found with a higher *α*_ME_ over the single‐phase counterparts, expanding the spectrum of multiferroic ME materials and the operating temperature range.^[^ ^19^ ^]^ A typical composite ME material is a layered combination of a FM and a FE material with phase interfaces. Various mechanisms including interfacial strain,^[^ ^20^ ^]^ interfacial charge,^[^ ^21^ ^]^ and exchange bias^[^ ^22^ ^]^ have been revealed and used to design multiferroic composite materials with a high *α*_ME_. Among these strategies, the interface strain has been investigated unremittingly and proved to be the most effective one. When subjected to an external magnetic field, the composite ME material generates mechanical strain in the FM phase due to the piezomagnetic/magnetostrictive effect. The mechanical strain mediated by the interface will introduce surface charges in the FE phase through the electrostrictive/piezoelectric effect (**Figure** **1**). The situation is reversed when subjected to an external electric field. In this regard, FM phases with large magnetostrictive effect and FE phases with large piezoelectric effects are preferred for constructing an interface with a high *α*_ME_. For example, strain‐mediated *α*_ME_ in CoFe_2_O_4_–BaTiO_3_ (CFO–BTO) core–shell structure can be ≈8.1 mV cm^−1^ Oe^−1^, which is 35 times larger than a bulk CFO–BTO composite.^[^ ^23^ ^]^ Core–shell structures such as La_0.7_Sr_0.3_MnO_3_–BaTiO_3_ (LSMO–BTO)^[^ ^24^ ^]^ and Pb(ZrTi)O_3_–NiFe_2_O_4_ (PZT–NF)^[^ ^25^ ^]^ exhibit even higher *α*_ME_, ≈54.5 and *α*_ME_ ≈ 195 mV cm^−1^ Oe^−1^, respectively. In contrast, the *α*_ME_ of single‐phase ME coupling multiferroic materials^[9a]^ usually ranges from ≈0.84 to ≈12 mV cm^−1^ Oe^−1^. It is noteworthy that the magnetic centers of most ME materials are highly active in the electrochemical reactions. For example, the CoFe_2_O_4_ in CoFe_2_O_4_–BaTiO_3_and La_0.7_Sr_0.3_MnO_3_ in La_0.7_Sr_0.3_MnO_3_–BaTiO_3_ are extensively investigated as OER/HER electrocatalysts.^[^ ^26^ ^]^ It is assumed that the magnetic centers in ME materials are mainly contributed by unfulfilled 3d orbitals of transition metals (Co, Fe, Mn, etc); meanwhile, these cations serve as the main active sites for adsorption/desorption and electron transfer in electrocatalytic reactions due to the unoccupied 3d‐orbitals. Therefore, ME coupling materials are promising electrocatalyst candidates, although they are not been well exploited so far.

**Figure 2:**
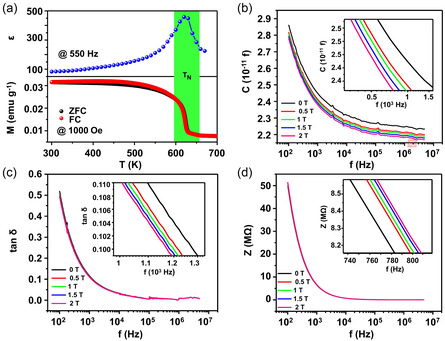
a) Temperature dependence of relative dielectric permittivity at frequency 550 Hz (top), and zero‐field‐cooling and field‐cooling plot in a temperature range of 300–700K with 1000 Oe magnetic field (bottom). b–d) Frequency dependence of capacitance (b), dielectric loss (c), and impedance (d) under different magnetic fields (0 ≤ *H* ≤ 2*T*) of (Bi_0.95_Nd_0.05_)(Fe_0.97_Mn_0.03_)O_3_ (BNFM) ceramics at room temperature. a–d) Reproduced with permission.^[^ ^27^ ^]^ Copyright 2017, AIP Publishing.

There are many techniques to verify the ME effect in a material. For example, the change of dielectric parameters with magnetic field variation confirms the strong ME coupling in (Bi_0.95_Nd_0.05_)(Fe_0.97_Mn_0.03_)O_3_ (BNFM) composite systems at room temperature.^[^ ^27^ ^]^ As shown in **Figure** **2**a, the relative dielectric permittivity (*ε*) exhibits significant changes near the Neel temperature (*T*_N_). With an increase in the magnetic field, the capacitance (*C*, Figure 2b) and dielectric loss (tan *δ*, Figure 2c) showed a systematic decrease, while impedance (*Z*, Figure 2d) increased. The change in these dielectric parameters was the most significant at 2T, which was attributed to the magnetic‐field‐induced magnetostriction behavior in the BNFM. Moreover, optical spectroscopy under various magnetic fields, including magnetic force microscopy, X‐ray magnetic circular dichroic photoelectron microscopy, and hysteresis loops obtained via piezoresponse force microscopy (PFM) at different magnetic field strengths, is applicable for characterizing the ME effect as well.^[^ ^28^ ^]^

## 3 Magnetoelectric Effect in Electrocatalysis

Although both the single‐phase and the composite ME materials have been investigated for a long time in the condensed matter physics, their (or component) catalytic activity was separately studied in the energy material field. Making use of the ME coupling effect in the catalytic materials to prompt their performance is a new topic yet to be fully studied. Several recent reports started to focus on the potential of using multiferroic catalysts to enhance electrocatalytic performance.

Qi et al.^[10c]^ synthesized Sr‐doped BiFeO_3_ (BFO) through a sol–gel method, which exhibits the coexistence of ferrimagnetism and ferroelectricity at room temperature. At the same time, such a multiferroic material also gives an HER activity ≈17.6 times higher than pure BFO. Similarly, Hums Khan et al.^[10b]^ synthesized an orthogonal multiferroic TbFeO_3_ (TFO), which exhibits high performance in both OER (Tafel slopes is 78.7 mV dec^−1^) and HER (Tafel slope is 63.5 mV dec^−1^).

**Figure 3:**
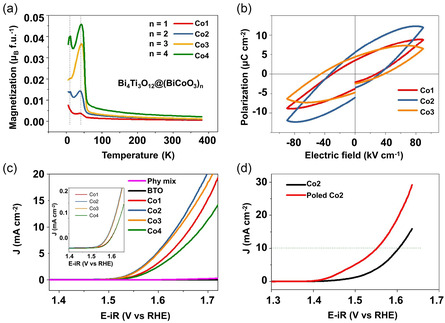
a) Temperature dependence of the magnetization *M–T* curves under a magnetic field of 500 Oe. b) *P–E* loops at the maximum electric field of 90 kV cm^−1^ measured at room temperature. c) Linear sweep voltammetry (LSV) curves were obtained at the scan rate of 5 mV s^−1^, and the inset shows the corresponding normalized curve based on the Brunauer–Emmett–Teller (BET) surface area. d) LSV curves of Co2 after corona poling. a–d) Reproduced under the terms of the CC‐BY Creative Commons Attribution 4.0 International license (https://creativecommons.org/licenses/by/4.0)^[10a]^ Copyright 2019, The Authors, published by Springer Nature.

In 2019, Li et al.^[10a]^ synthesized a heterojunction Bi_4_Ti_3_O_12_@(BiCoO_3_)_n_ (BCTO, and *n* = 1, 2, 3, 4 denoted as Co1, Co2, Co3, Co4). This kind of in situ‐grown heterojunction is a kind of composite multiferroic material with a possible ME coupling effect. The materials were confirmed with the coexistence of antiferromagnetic (AFM) and FE ordering by the magnetization–temperature (*M–T*, **Figure** **3**a) and polarization–electric field (*P–E*, Figure 3b) curves. It showed excellent OER performance, as evidenced by the higher current density (Figure 3c). Compared to a physical mixture of Bi_4_Ti_3_O_12_ and BiCoO_3_ (physical mix in Figure 3c) which has no interface for possible couplings, the enhanced performance of BCTO was attributed to the synergistic effect of the regulated magnetic (spin/electronic regulation) and ferroelectric (polarization) properties. The FE polarization provides additional driving forces toward charge transport on the electrode due to band bending and attracts more charged interactants. In addition, the polarization inside the catalysts was enhanced further by aligning the random ferroelectric polarization under a corona polar method, leading to a further improved OER performance (Figure 3d).

**Figure 4:**
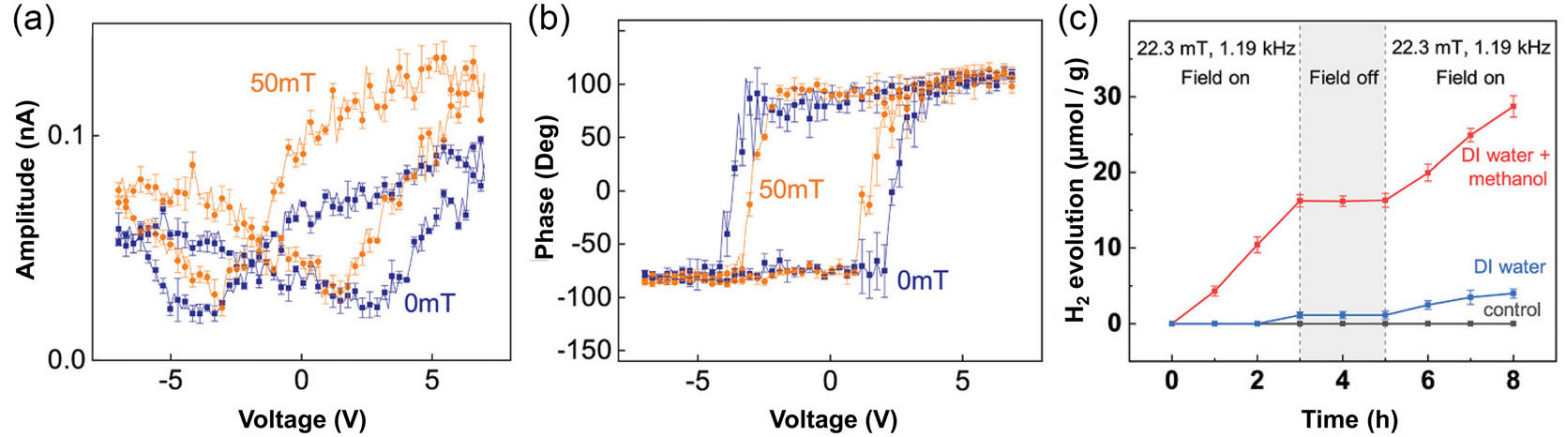
a,b) Magnetic‐field‐dependent local PFM hysteresis: a) amplitude loops and b) phase loops. c) Hydrogen evolution measured from gas chromatography (GC) spectra under a magnetic field as a function of time. Control samples indicate DI water, methanol/DI water solution, CFO nanoparticles mixed with DI water, and CFO nanoparticles mixed with methanol/DI water solution. a–c) Reproduced with permission.^[^ ^28^ ^]^ Copyright 2022, Wiley‐VCH.

**Figure 5:**
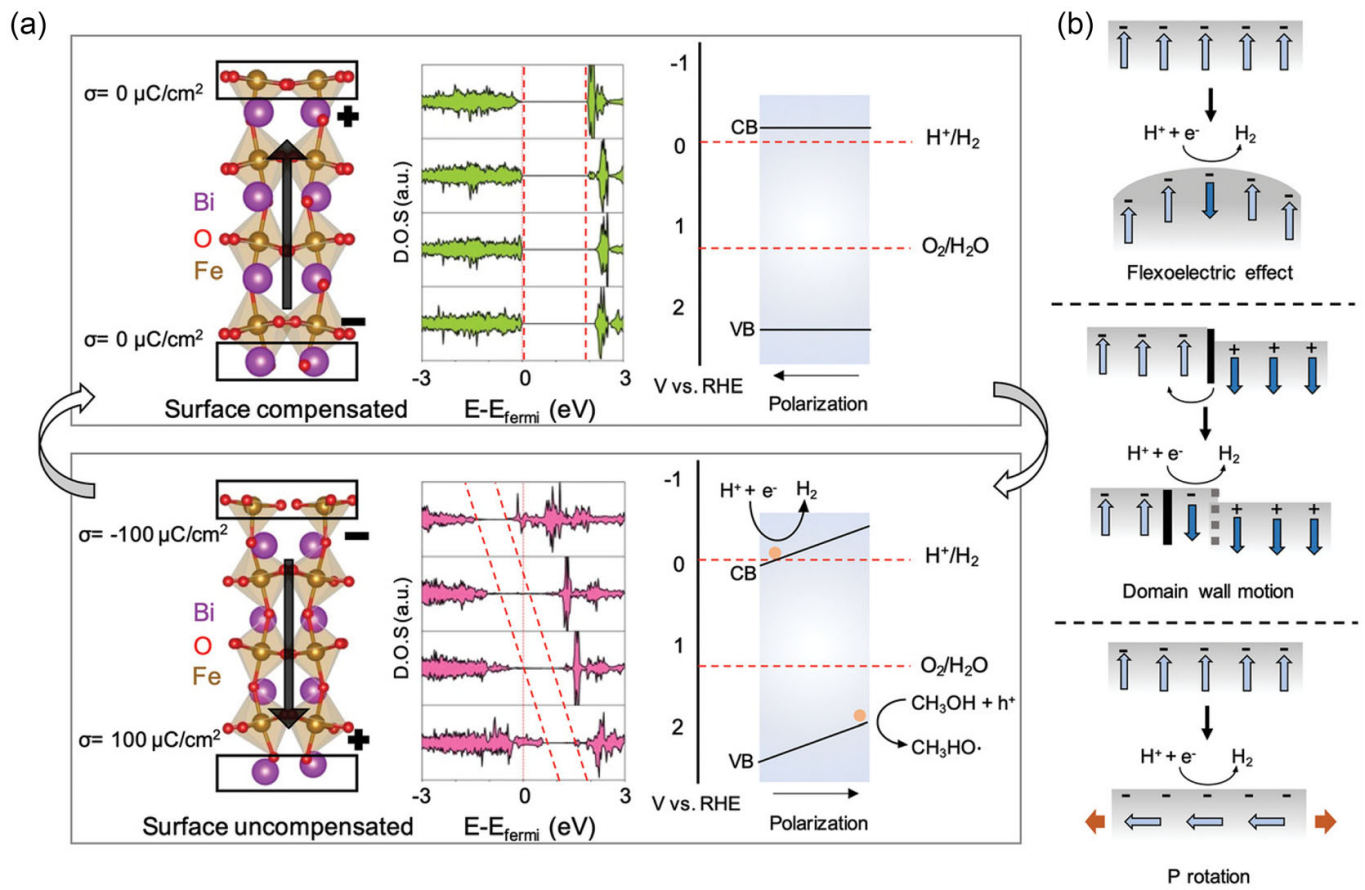
a) Calculated BFO (001) slabs (left) with a layer‐by‐layer density of states (middle), and the schematic of band bending caused by the reversal of the polarization (right) for up‐polarization (surface compensated, top) and down‐polarization (surface uncompensated, bottom). b) Possible mechanisms for the polarization reversal by ME coupling in core–shell nanoparticles. Strain leads to a flexoelectric effect (top), the strain‐induced motion of domain walls (middle), and strain‐induced in‐plane polarization rotation (bottom). a,b) Reproduced with permission.^[^ ^28^ ^]^ Copyright 2022, Wiley‐VCH.

In 2022, Donghoon Kim et al.^[^ ^28^ ^]^ reported on the application of a CoFe_2_O_4_–BiFeO_3_ (CFO–BFO) core–shell nanoparticle as a composite ME multiferroic material for HER. CFO with a high magnetostrictive coefficient was the magnetic core and a single‐phase multiferroic material BFO was the shell. The PFM (**Figure** **4**) indicated that the piezoelectric effect of the CFO–BFO nanoparticles was dependent on the magnetic field. An external magnetic field of 50 mT produced an electric field of 16.25 MV m^−1^, while the piezoelectric response significantly increased when the magnetic field was applied. Moreover, the HER performance of CFO nanoparticles, CFO + BFO physical mixture nanoparticles in deionized (DI) water, and DI water/methanol solution was tested with and without a 22.3 mT external magnetic field. No hydrogen was observed during the reaction when the CFO + BFO physical mixture was used as a catalyst, regardless of the presence of a magnetic field. However, when the core–shell structure samples were used as catalysts, hydrogen production increased with an external magnetic field. Through density functional theory (DFT) calculations (**Figure** **5**), the researchers found that the charge of the BFO (001) oriented slab is unstable and composed of positive and negative charges. When an external magnetic field was applied, the magnetic core underwent magnetostriction and transferred the strain stress to the shell, inducing a piezoelectric effect that reversed the direction of FE polarization, resulting in positive and negative charges on the surfaces of Bi^3+^O^2−^ and Fe^3+^O^2−^_2_, respectively. This created an unsaturated surface with severe band bending, generating an electric charge on the catalyst surface that facilitated hydrogen production during the HER reaction. With those limited but convincing reports, it can be concluded that the ME effect can be taken as a new and promising way for the development of novel electrocatalysts.

## 4 Conclusion and Outlook

**Figure 6:**
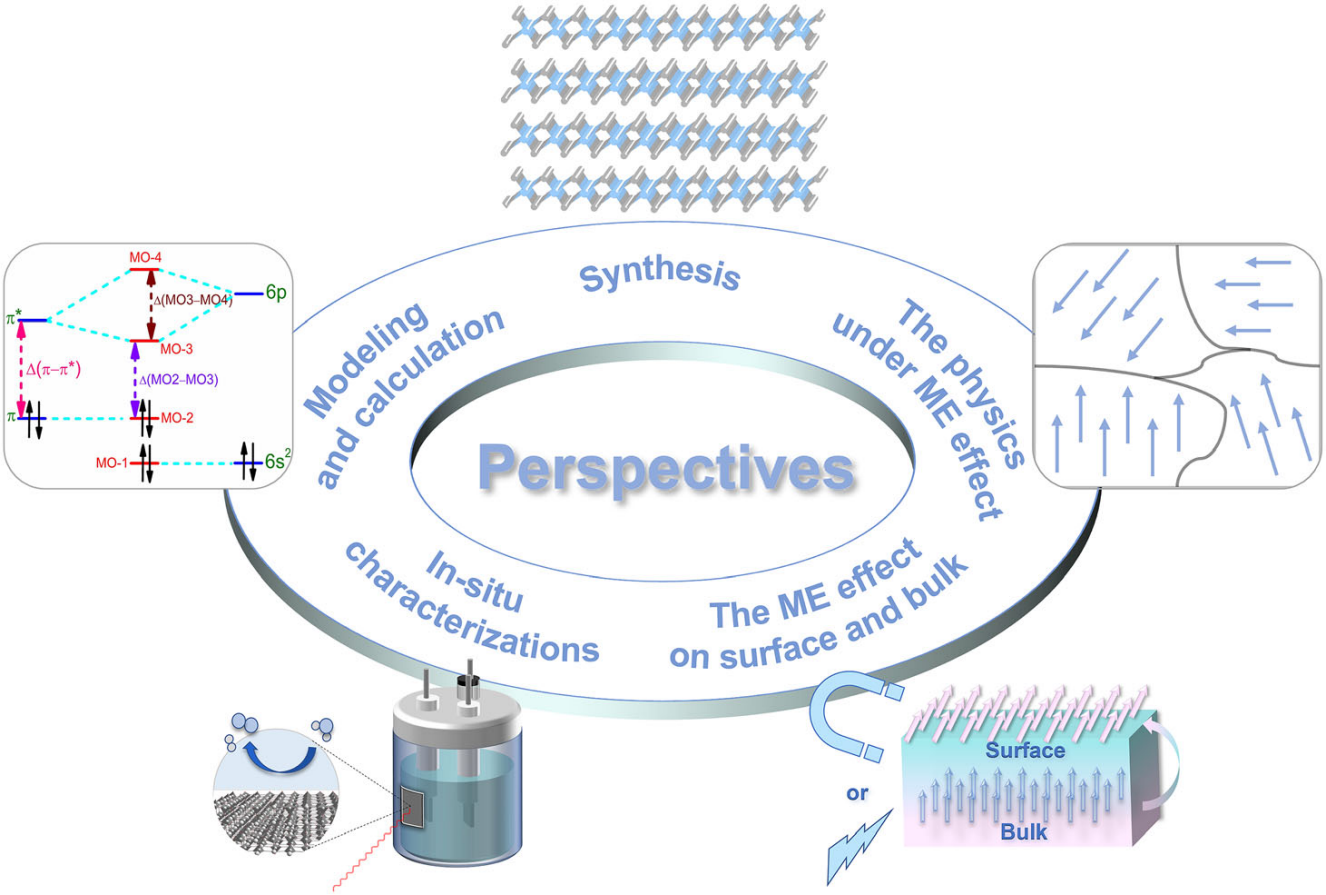
Perspectives to further study the ME effects in electrocatalysis.

This perspective briefly discusses the potential of using ME coupling materials in electrocatalysis. The electronic structure of these materials can be tuned by applying an external magnetic or electric field in theory, which could result in the improved performance by facilitating the electron behaviors during the catalytic reactions. Based on the limited reports above, the following perspectives are provided for further study to fully unleash the potential of the ME effect in electrocatalysis (**Figure** **6**).

First, more methodologies for synthesizing multiferroic ME materials with electrocatalytic activity must be developed. Relying on interface engineering and technologies, various materials with strong ME coupling at room temperature can be synthesized by complicated and costly routes currently.^[^ ^29^ ^]^ However, further research is needed to develop simple and low‐cost methods for synthesizing ME electrocatalysts, which have additional requirements on the morphology and surface area.

Second, the underlying physics and the real role played in the catalysis of ME coupling are far from clear. As we all know, ME coupling is significantly affected by domain structure, domain wall dynamics, and spin‐lattice dynamics. These can be probed by factors such as magnetic force microscopy, magneto‐optical Kerr rotation, and X‐ray magnetic circular dichroism photoelectron microscopy, although, most of these techniques show limitations when incorporated with electrochemical setups. In this regard, theoretical studies such as micromagnetic simulation are proposed to be applied to studies such as the internal magnetic domain structure and domain wall dynamics of multiferroic materials. Some studies^[^ ^30^ ^]^ have demonstrated that micromagnetic simulations could simulate domain structure and confirm the magnetization rotation that occurs due to the strain‐mediated ME interaction.

Third, the surface and near‐surface regions of electrocatalysts play an important role in electrocatalytic reactions. The difference between the surface and the bulk ME effect is often ignored in the study of multiferroic materials. The strengths of the ME effect on the surface and bulk can be described in the ME coupling coefficient *α*.^[^ ^31^ ^]^ Nevertheless, establishing a correlation between the surface and bulk ME effect through *α* remains an enormous challenge because of the different ME coupling mechanisms between the surface and bulk ME effect. It will be helpful to construct theoretical models of surface and bulk under consideration of more physical parameters such as magnetism and surface charge.^[^ ^32^ ^]^ Alternatively, the characterization of these physical parameters can be carried out by factors such as spin‐polarized scanning tunneling microscopy.^[^ ^33^ ^]^ However, such application in electrocatalysis is still limited by the difficulty of operando measurement under electrochemical conditions.

Fourth, in situ structural characterizations can lead to a better understanding of the ME effect by revealing dynamic atomic structural details under operando conditions. This is especially important for materials that show structural flexibility in OER and may undergo surface reconstruction.^[7c]^ It is desirable to incorporate X‐ray absorption spectroscopy (XAS), X‐ray photoelectron spectroscopy (XPS), and transmission electron microscope (TEM) with electrochemical setup to carry out the operando measurement. The development of related in situ techniques is important but challenging.

Fifth, DFT^[^ ^34^ ^]^ studies play an important role in the investigation of the ME effect. It can be applied to construct slab models for the study of the surface ME effect as mentioned above.^[^ ^28^ ^]^ Besides, it also can be applied to study ME‐related physical properties such as spin polarization and spin orientation under the effect of ME coupling. In this regard, DFT calculations with consideration of spin are important in investigating the spin magnetic moments and the magnetism of itinerant electrons in solid‐state materials. For example, spin‐polarized DFT calculation was applied to simulate the spin channel of ferromagnetic catalysts, which has been proven critical for efficient triplet O_2_ turnover.^[^ ^8^ ^]^ With the help of DFT studies, more fundamentals regarding the ME effect will be revealed and to be associated with the electrocatalytic activity of multiferroic catalysts. This will greatly facilitate the design of suitable ME multiferroic materials for high‐performing electrocatalysis.

## Conflict of Interest

The authors declare no conflict of interest.
